# Safety of Bariatric Surgery in ≥ 65-Year-Old Patients During the COVID-19 Pandemic

**DOI:** 10.1007/s11695-022-06067-z

**Published:** 2022-05-05

**Authors:** Rishi Singhal, Islam Omar, Brijesh Madhok, Yashasvi Rajeev, Yitka Graham, Abd A. Tahrani, Christian Ludwig, Tom Wiggins, Kamal Mahawar, On behalf of the GENEVA Collaborators, Michał Pędziwiatr, Piotr Major, Piotr Zarzycki, Athanasios Pantelis, Dimitris P. Lapatsanis, Georgios Stravodimos, Chris Matthys, Marc Focquet, Wouter Vleeschouwers, Antonio G. Spaventa, Carlos Zerrweck, Antonio Vitiello, Giovanna Berardi, Mario Musella, Alberto Sanchez-Meza, Felipe J. Cantu, Fernando Mora, Marco A. Cantu, Abhishek Katakwar, D. Nageshwar Reddy, Haitham Elmaleh, Mohammad Hassan, Abdelrahman Elghandour, Mohey Elbanna, Ahmed Osman, Athar Khan, Laurent Layani, Nalini Kiran, Andrey Velikorechin, Maria Solovyeva, Hamid Melali, Shahab Shahabi, Ashish Agrawal, Apoorv Shrivastava, Ankur Sharma, Bhavya Narwaria, Mahendra Narwaria, Asnat Raziel, Nasser Sakran, Sergio Susmallian, Levent Karagöz, Murat Akbaba, Salih Zeki Pişkin, Ahmet Ziya Balta, Zafer Senol, Emilio Manno, Michele Giuseppe Iovino, Ahmed Osman, Mohamed Qassem, Sebastián Arana-Garza, Heitor P. Povoas, Marcos Leão Vilas-Boas, Alan Li, Basil J. Ammori, Hany Balamoun, Mohammed Salman, Amrit Manik Nasta, Ramen Goel, Hugo Sánchez-Aguilar, Miguel F. Herrera, Adel Abou-Mrad, Lucie Cloix, Guilherme Silva Mazzini, Leonardo Kristem, Andre Lazaro, Jose Campos, Joaquín Bernardo, Jesús González, Carlos Trindade, Octávio Viveiros, Rui Ribeiro, David Goitein, David Hazzan, Lior Segev, Tamar Beck, Hernán Reyes, Jerónimo Monterrubio, Paulina García, Marine Benois, Radwan Kassir, Alessandro Contine, Moustafa Elshafei, Sueleyman Aktas, Sylvia Weiner, Till Heidsieck, Luis Level, Silvia Pinango, Patricia Martinez Ortega, Rafael Moncada, Victor Valenti, Ivan Vlahović, Zdenko Boras, Arnaud Liagre, Francesco Martini, Gildas Juglard, Manish Motwani, Sukhvinder Singh Saggu, Hazem Al Momani, Luis Adolfo Aceves López, María Angelina Contreras Cortez, Rodrigo Aceves Zavala, Christine D’Haese, Ivo Kempeneers, Jacques Himpens, Andrea Lazzati, Luca Paolino, Sarah Bathaei, Abdulkadir Bedirli, Aydın Yavuz, Çağrı Büyükkasap, Safa Özaydın, Andrzej Kwiatkowski, Katarzyna Bartosiak, Maciej Walędziak, Antonella Santonicola, Luigi Angrisani, Paola Iovino, Rossella Palma, Angelo Iossa, Cristian Eugeniu Boru, Francesco De Angelis, Gianfranco Silecchia, Abdulzahra Hussain, Srivinasan Balchandra, Izaskun Balciscueta Coltell, Javier Lorenzo Pérez, Ashok Bohra, Altaf K. Awan, Brijesh Madhok, Paul C. Leeder, Sherif Awad, Waleed Al-Khyatt, Ashraf Shoma, Hosam Elghadban, Sameh Ghareeb, Bryan Mathews, Marina Kurian, Andreas Larentzakis, Gavriella Zoi Vrakopoulou, Konstantinos Albanopoulos, Ahemt Bozdag, Azmi Lale, Cuneyt Kirkil, Mursid Dincer, Ahmad Bashir, Ashraf Haddad, Leen Abu Hijleh, Bruno Zilberstein, Danilo Dallago de Marchi, Willy Petrini Souza, Carl Magnus Brodén, Hjörtur Gislason, Kamran Shah, Antonio Ambrosi, Giovanna Pavone, Nicola Tartaglia, S. Lakshmi Kumari Kona, K. Kalyan, Cesar Ernesto Guevara Perez, Miguel Alberto Forero Botero, Adrian Covic, Daniel Timofte, Madalina Maxim, Dashti Faraj, Larissa Tseng, Ronald Liem, Gürdal Ören, Evren Dilektasli, Ilker Yalcin, Hudhaifa AlMukhtar, Mohammed Al Hadad, Rasmi Mohan, Naresh Arora, Digvijaysingh Bedi, Claire Rives-Lange, Jean-Marc Chevallier, Tigran Poghosyan, Hugues Sebbag, Lamia Zinaï, Saadi Khaldi, Charles Mauchien, Davide Mazza, Georgiana Dinescu, Bernardo Rea, Fernando Pérez-Galaz, Luis Zavala, Anais Besa, Anna Curell, Jose M. Balibrea, Carlos Vaz, Luis Galindo, Nelson Silva, José Luis Estrada Caballero, Sergio Ortiz Sebastian, João Caetano Dallegrave Marchesini, Ricardo Arcanjo da Fonseca Pereira, Wagner Herbert Sobottka, Felipe Eduardo Fiolo, Matias Turchi, Antonio Claudio Jamel Coelho, Andre Luis Zacaron, André Barbosa, Reynaldo Quinino, Gabriel Menaldi, Nicolás Paleari, Pedro Martinez-Duartez, Gabriel Martínez de Aragon Ramírez de Esparza, Valentin Sierra Esteban, Antonio Torres, Jose Luis Garcia-Galocha, MIguel Josa, Jose Manuel Pacheco-Garcia, Maria Angeles Mayo-Ossorio, Pradeep Chowbey, Vandana Soni, Hercio Azevedo de Vasconcelos Cunha, Michel Victor Castilho, Rafael Meneguzzi Alves Ferreira, Thiago Alvim Barreiro, Alexandros Charalabopoulos, Elias Sdralis, Spyridon Davakis, Benoit Bomans, Giovanni Dapri, Koenraad Van Belle, MazenTakieddine, Pol Vaneukem, Esma Seda Akalın Karaca, Fatih Can Karaca, Aziz Sumer, Caghan Peksen, Osman Anil Savas, Elias Chousleb, fahad Elmokayed, Islam Fakhereldin, Hany Mohamed Aboshanab, Talal Swelium, Ahmad Gudal, Lamees Gamloo, Ayushka Ugale, Surendra Ugale, Clara Boeker, Christian Reetz, Ibrahim Ali Hakami, Julian Mall, Andreas Alexandrou, Efstratia Baili, Zsolt Bodnar, Almantas Maleckas, Rita Gudaityte, Cem Emir Guldogan, Emre Gundogdu, Mehmet Mahir Ozmen, Deepti Thakkar, Nandakishore Dukkipati, Poonam Shashank Shah, Shashank Subhashchandra Shah, Simran Shashank Shah, Md Tanveer Adil, Periyathambi Jambulingam, Ravikrishna Mamidanna, Douglas Whitelaw, Md Tanveer Adil, Vigyan Jain, Deepa Kizhakke Veetil, Randeep Wadhawan, Antonio Torres, Max Torres, Tabata Tinoco, Wouter Leclercq, Marleen Romeijn, Kelly van de Pas, Ali K. Alkhazraji, Safwan A. Taha, Murat Ustun, Taner Yigit, Aatif Inam, Muhammad Burhanulhaq, Abdolreza Pazouki, Foolad Eghbali, Mohammad Kermansaravi, Amir Hosein Davarpanah Jazi, Mohsen Mahmoudieh, Neda Mogharehabed, Gregory Tsiotos, Konstantinos Stamou, Francisco J. Barrera Rodriguez, Marco A. Rojas Navarro, Omar MOhamed Torres, Sergio Lopez Martinez, Elda Rocio Maltos Tamez, Gustavo A. Millan Cornejo, Jose Eduardo Garcia Flores, Diya Aldeen Mohammed, Mohamad Hayssam Elfawal, Asim Shabbir, Kim Guowei, Jimmy By So, Elif Tuğçe Kaplan, Mehmet Kaplan, Tuğba Kaplan, DangTuan Pham, Gurteshwar Rana, Mojdeh Kappus, Riddish Gadani, Manish Kahitan, Koshish Pokharel, Alan Osborne, Dimitri Pournaras, James Hewes, Errichetta Napolitano, Sonja Chiappetta, Vincenzo Bottino, Evelyn Dorado, Axel Schoettler, Daniel Gaertner, Katharina Fedtke, Francisco Aguilar-Espinosa, Saul Aceves-Lozano, Alessandro Balani, Carlo Nagliati, Damiano Pennisi, Andrea Rizzi, Francesco Frattini, Diego Foschi, Laura Benuzzi, Chirag Parikh, Harshil Shah, Enrico Pinotti, Mauro Montuori, Vincenzo Borrelli, Jerome Dargent, Catalin A Copaescu, Ionut Hutopila, Bogdan Smeu, Bart Witteman, Eric Hazebroek, Laura Deden, Laura Heusschen, Sietske Okkema, Theo Aufenacker, Willem den Hengst, Wouter Vening, Yonta van der Burgh, Ahmad Ghazal, Hamza Ibrahim, Mourad Niazi, Bilal Alkhaffaf, Mohammad Altarawni, Giovanni Carlo Cesana, Marco Anselmino, Matteo Uccelli, Stefano Olmi, Christine Stier, Tahsin Akmanlar, Thomas Sonnenberg, Uwe Schieferbein, Alejandro Marcolini, Diego Awruch, Marco Vicentin, Eduardo Lemos de Souza Bastos, Samuel Azenha Gregorio, Anmol ahuja, Tarun mittal, Roel Bolckmans, Tom Wiggins, Clément Baratte, Judith Aron Wisnewsky, Laurent Genser, Lynn Chong, Lillian Taylor, Salena Ward, Lynn Chong, Lillian Taylor, Michael W Hi, Helen Heneghan, Naomi Fearon, Andreas Plamper, Karl Rheinwalt, Helen Heneghan, Justin Geoghegan, Kin Cheung Ng, Naomi Fearon, Krzysztof Kaseja, Maciej Kotowski, Tarig A Samarkandy, Adolfo Leyva-Alvizo, Lourdes Corzo-Culebro, Cunchuan Wang, Wah Yang, Zhiyong Dong, Manel Riera, Rajesh Jain, Hosam Hamed, Mohammed Said, Katia Zarzar, Manuel Garcia, Ahmet Gökhan Türkçapar, Ozan Şen, Edoardo Baldini, Luigi Conti, Cacio Wietzycoski, Eduardo Lopes, Tadeja Pintar, Jure Salobir, Cengiz Aydin, Semra Demirli Atici, Anıl Ergin, Huseyin Ciyiltepe, Mehmet Abdussamet Bozkurt, Mehmet Celal Kizilkaya, Nezihe Berrin Dodur Onalan, Mariana Nabila Binti Ahmad Zuber, Wei Jin Wong, Amador Garcia, Laura Vidal, Marc Beisani, Jorge Pasquier, Ramon Vilallonga, Sharad Sharma, Chetan Parmar, Lyndcie Lee, Pratik Sufi, Hüseyin Sinan, Mehmet Saydam

**Affiliations:** 1 Upper GI unit, Birmingham Heartlands Hospital, University Hospital Birmingham NHS Foundation Trust, Bordesley Green East, Birmingham, West Midlands B9 5SS UK; 2 Healthier Weight, Birmingham, UK; 3 General Surgery Department, Wirral University Teaching Hospital NHS Foundation Trust, North West, Wirral, UK; 4 Upper GI unit, University Hospital of Derby and Burton NHS Foundation Trust, East Midlands, Derby, UK; 5 Pediatric Accidents and Emergencies Department, London Northwest University Healthcare NHS Trust, London, UK; 6 Faculty of Health Sciences and Wellbeing, University of Sunderland, North East, Sunderland, UK; 7 Faculdad de Pyscologia, Universidad Anahuac, Anahuac, Naucalpan de Juárez, Mexico; 8 Institute of Metabolism and Systems Research, College of Medical and Dental Sciences, University of Birmingham, Birmingham, UK; 9 Centre for Endocrinology, Diabetes, and Metabolism, Birmingham Health Partners, Birmingham, UK; 10 Department of Diabetes and Endocrinology, University Hospitals Birmingham NHS Foundation Trust, Birmingham, West Midlands UK; 11 Institute of Metabolism and Systems Research, College of Medical and Dental Sciences, University of Birmingham, Birmingham, West Midlands UK; 12 Bariatric Unit, South Tyneside and Sunderland NHS Foundation Trust, North East, Sunderland, UK

**Keywords:** Obesity, Older patients, SARS-CoV-2, Resuming elective surgery, Metabolic surgery

## Abstract

**Background:**

Age ≥ 65 years is regarded as a relative contraindication for bariatric surgery. Advanced age is also a recognised risk factor for adverse outcomes with Coronavirus Disease-2019 (COVID-19) which continues to wreak havoc on global populations. This study aimed to assess the safety of bariatric surgery (BS) in this particular age group during the COVID-19 pandemic in comparison with the younger cohort.

**Methods:**

We conducted a prospective international study of patients who underwent BS between 1/05/2020 and 31/10/2020. Patients were divided into two groups — patients ≥ 65-years-old (Group I) and patients < 65-years-old (Group II). The two groups were compared for 30-day morbidity and mortality.

**Results:**

There were 149 patients in Group 1 and 6923 patients in Group II. The mean age, preoperative weight, and BMI were 67.6 ± 2.5 years, 119.5 ± 24.5 kg, and 43 ± 7 in Group I and 39.8 ± 11.3 years, 117.7±20.4 kg, and 43.7 ± 7 in Group II, respectively. Approximately, 95% of patients in Group 1 had at least one co-morbidity compared to 68% of patients in Group 2 (*p* = < 0.001).

The 30-day morbidity was significantly higher in Group I (11.4%) compared to Group II (6.6%) (*p* = 0.022). However, the 30-day mortality and COVID-19 infection rates were not significantly different between the two groups.

**Conclusions:**

Bariatric surgery during the COVID-19 pandemic is associated with a higher complication rate in those ≥ 65 years of age compared to those < 65 years old. However, the mortality and postoperative COVID-19 infection rates are not significantly different between the two groups.

**Graphical abstract:**

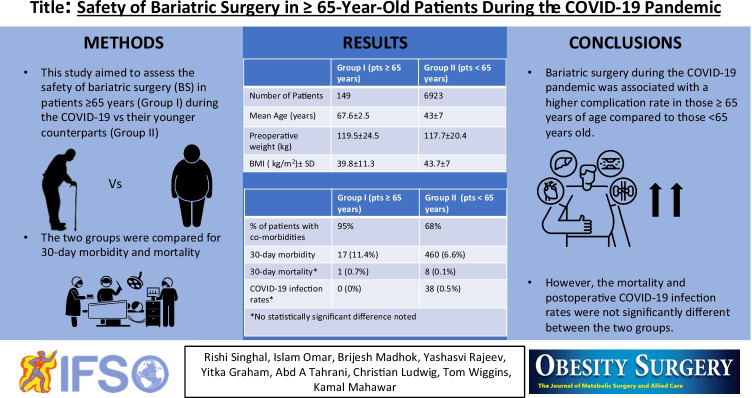

## Introduction

Bariatric surgery is currently the only evidence-based durable treatment option for patients with obesity and related comorbidities. An ageing population worldwide presents a challenge to all healthcare practitioners, including those involved in providing obesity management services [1, 2]. Previously, advanced age was considered a relative contraindication for bariatric surgery [3]. However, the evolution of laparoscopic techniques and advances in perioperative care protocols have changed perceptions [4, 5].

While some studies confirm good weight loss and acceptable postoperative morbidity and mortality in older individuals, others show significant perioperative morbidity and mortality with varying weight loss results [6–8]. Additionally, the heterogeneity of the studies with different age cutoff points and definitions of the older patients’ population prevents the generalisation of these results [9, 10].

Older age is associated with an unfavourable prognosis with COVID-19 should a patient undergoing bariatric surgery develop perioperative COVID-19 infection. At the same time, obesity and its associated comorbidities also increase the risk of adverse outcomes with COVID-19 [11–13]. This poses a dilemma for healthcare professionals dealing with older patients seeking bariatric surgery.

The present study aimed to understand the safety of bariatric surgery in ≥ 65-years-old patients during the COVID-19 pandemic. This study was a subset analysis of the GENEVA dataset; a global study aimed to prospectively assess the safety of bariatric surgery during the COVID-19 pandemic [14–16].

## Methods

### Study Design, Setting, and Population

The GENEVA study was a global, multicentre, observational study of Bariatric Surgery (elective primary, elective revisional, and emergency) performed between 1/05/2020 and 31/10/2020 in the adult (≥ 18 years) population. The detailed methods have been described elsewhere [14–16].

We used 65 years as a cutoff point to define the older age group as per the World Health Organisation and The National Institute for Health and Care Excellence (NICE) definitions [17, 18]. We divided patients undergoing primary BS into two groups — those ≥ 65 years old (Group I) and those < 65 years old (Group II). The two groups were compared with each other with regard to basic demographics, 30-day morbidity and mortality, postoperative symptomatic COVID-19 infection rates, and procedure choice.

The main outcome measures of this study were 30-day all-cause and COVID-19 specific morbidity and mortality. Continuous data were presented as mean ± standard deviation (SD) or median (IQR) depending on data distribution. Frequencies were used to summarise categorical variables. Independent *t*-test or Mann Whitney U test examined differences between continuous variables depending on data distribution. A chi-square test or Fisher’s exact test was used to compare categorical variables. Significance levels were set at *p* < 0.05. Statistical analysis was performed using the Statistical Package for the Social Sciences (SPSS) statistical software, version 27.0 (SPSS Inc).

## Results

Data were collected from 179 centres in 42 countries by 470 surgeons (Appendix 1). Seven thousand ninety-two adult patients who underwent primary BS between 01/05/2020 and 31/10/2020 were included. Complete 30-day morbidity and mortality data were available for 7084 (99.88%) patients. The mean age of the entire cohort was 40.35 ± 11.9 years, and 5197 (73.4%) were females. The mean preoperative weight and body mass index (BMI) was 119.49 ± 24.4 Kg and 43.03 ± 6.9 Kg/m^2^, respectively.

Table 1 compares the demographics of the two groups. The mean age for group I was 67.6 ± 2.5 years, and for group II was 39.8 ± 11.3 years. Group I included more patients of white ethnicity (84.6%) than Group II (74.3%) (*p* = 0.004). The rest of the demographic parameters, including pre-operative BMI and weight, were comparable between the two groups (Table 1).

**Table 1:** Comparison between the two groups according to demographics

	Group I (≥ 65) (*n* =149#)	Group II (˂ 65) (*n* =6923#)	*χ*^2^	*p*
Age				
Min.–max.	65–76	17–64		
Mean ± SD	67.6 ± 2.5	39.8 ± 11.3		
Sex				
Female	102 (68.5%)	5085 (73.5%)	1.869	0.172
Male	47 (31.5%)	1837 (26.5%)		
Preoperative Weight (Kg)				
Min.–max.	52–268	72–178	U = 506083.0	0.695
Mean ± SD	119.5 ± 24.5	117.7 ± 20.4		
Calculated Preoperative BMI				
Min.–max.	18–100.6	29–68.7	t = 1.208	0.227
Mean ± SD	43 ± 7	43.7 ± 7		
White vs non white				
No	23 (15.4%)	1780 (25.7%)	8.107^*^	0.004^*^
Yes	126 (84.6%)	5143 (74.3%)		
Ethnicity of patient				
I, American Indian or Alaska Native	0 (0%)	10 (0.1%)		
II, Asian	8 (5.4%)	390 (5.6%)		
III, Black or African American	1 (0.7%)	86 (1.2%)		
IV, Hispanic or Latino	14 (9.4%)	1280 (18.5%)		
V, Native Hawaiian or Other Pacific Islander	0 (0%)	14 (0.2%)		
VI, White	126 (84.6%)	5143 (74.3%)		

χ^2^: Chi-square test; p: *p*-value for comparing the two studied groups; *Statistically significant at p ≤ 0.05; #Cases with missing data were excluded from the comparison between the two age groups; **#***p*-value excluded *missing data* from comparing the studied groups

I: American Indian or Alaska Native. A person having origins in any of the original peoples of North and South America (including Central America), and who maintains tribal affiliation or community attachment

II: Asian. A person having origins in any of the original peoples of the Far East, Southeast Asia, or the Indian subcontinent including, for example, Cambodia, China, India, Japan, Korea, Malaysia, Pakistan, the Philippine Islands, Thailand, and Vietnam

III: Black or African American. A person having origins in any of the black racial groups of Africa. Terms such as ‘Haitian’ or ‘Negro’ can be used in addition to ‘Black or African American’

IV: Hispanic or Latino. A person of Cuban, Mexican, Puerto Rican, South or Central American, or other Spanish culture or origin, regardless of race. The term, ‘Spanish origin’, can be used in addition to ‘Hispanic or Latino’.

V: Native Hawaiian or Other Pacific Islander. A person having origins in any of the original peoples of Hawaii, Guam, Samoa, or other Pacific Islands

VI: White. A person having origins in any of the original peoples of Europe, the Middle East, or North Africa

Table 2 details the prevalence of comorbidities and smoking status in the two groups. Nearly 95% of patients in Group I had at least one co-morbidity compared to 68% of patients in Group II (*p* = < 0.001). Specifically, a significantly greater proportion of patients in Group I suffered from diabetes mellitus (DM) (48.3% vs 20.2%), hypertension (74.5% vs 29.9%), obstructive sleep apnoea requiring continuous positive airway pressure (CPAP) therapy (25.5% vs 13.1%), and hypercholesterolemia (41.6% vs 21.1%) compared to Group II (all comparisons *p* = < 0.001) (Table 2). In Group II, 14.8% of patients were current smokers, compared to 7.4% of Group I (*p* = <0.001).

**Table 2:** Comparison between the two groups according to comorbidity and smoking status

	Group I (≥ 65)	Group II (˂ 65)	*χ*^2^	*p*
Any comorbidity	(*n* =149)	(*n* =6923)		
No	8 (5.4%)	2193 (31.7%)	47.093^*^	<0.001^*^
Yes	141 (94.6%)	4730 (68.3%)		
Type 2 diabetes not on medication	(*n* =149)	(*n* =6923)		
No	144 (96.6%)	6507 (94%)	1.834	0.176
Yes	5 (3.4%)	416 (6%)		
Type 2 diabetes on oral medication	(*n* =149)	(*n* =6923)		
No	98 (65.8%)	6116 (88.3%)	69.708^*^	< 0.001^*^
Yes	51 (34.2%)	807 (11.7%)		
Type 2 diabetes on insulin)	(*n* =149)	(*n* =6923)		
No	126 (84.6%)	6694 (96.7%)	62.438^*^	< 0.001^*^
Yes	23 (15.4%)	229 (3.3%)		
Overall diabetes	(*n* =149)	(*n* =6923)		
No	77 (51.7%)	5524 (79.8%)	69.983^*^	< 0.001^*^
Yes	72 (48.3%)	1399 (20.2%)		
Hypertension	(*n* =149)	(*n* =6923)		
No	38 (25.5%)	4851 (70.1%)	135.764^*^	< 0.001^*^
Yes	111 (74.5%)	2072 (29.9%)		
Sleep apnea not on CPAP	(*n* =149)	(*n* =6923)		
No	126 (84.6%)	6091 (88%)	1.604	0.205
Yes	23 (15.4%)	832 (12%)		
Sleep apnea on CPAP	(*n* =149)	(*n* =6923)		
No	111 (74.5%)	6014 (86.9%)	19.254^*^	< 0.001^*^
Yes	38 (25.5%)	909 (13.1%)		
Hypercholesterolemia	(*n* =149)	(*n* =6923)		
No	87 (58.4%)	5461 (78.9%)	36.233^*^	< 0.001^*^
Yes	62 (41.6%)	1462 (21.1%)		
Other comorbidities	(*n* =149)	(*n* =6923)		
No	87 (58.4%)	4926 (71.2%)	11.516^*^	0.001^*^
Yes	62 (41.6%)	1997 (28.8%)		
Smoking status	(*n* =149)	(*n* =6922^#^)		
Current smoker	11 (7.4%)	1027 (14.8%)	28.300^*^	< 0.001^*^
Ex-smoker	40 (26.8%)	887 (12.8%)		
Non-smoker	98 (65.8%)	5008 (72.3%)		

χ^2^: Chi-square test; FE: Fisher Exact; p: *p*-value for comparing the two studied groups; *Statistically significant at *p* ≤ 0.05; #Cases with missing data were excluded from the comparison between the two groups

*CPAP*, continuous positive airway pressure

The most common operation type in both groups was laparoscopic sleeve gastrectomy (LSG) (Group 1: 51.0%; Group 2: 56.4%). This was followed by Roux-en-Y gastric bypass (RYGB) (Group 1: 32.9%; Group 2: 29.4%) and one-anastomosis gastric bypass (OAGB) (Group 1: 8.7%; Group 2: 10.0%). Other forms of procedures were performed in 7.4% (Group 1) and 4.2% (Group 2) of individuals. There were no significant differences in procedure choice between the two groups (*p* = 0.164) (Table 3).

**Table 3:** Comparison between the two age groups according to the surgical procedures

	Group I (≥ 65) (*n* =149)	Group II (˂ 65) (*n* =6923)	*χ*^2^	*p*
Surgical procedure				
LSG	76 (51%)	3907 (56.4%)	5.111	0.164
RYGB	49 (32.9%)	2038 (29.4%)		
OAGB	13 (8.7%)	689 (10%)		
Others	11 (7.4%)	289 (4.2%)		

χ^2^: Chi-square test; p: *p*-value for comparing between the two studied groups; *Statistically significant at *p* ≤ 0.05

*LSG*, laparoscopic sleeve gastrectomy; *OAGB*, one anastomosis gastric bypass; *RYGB*, Roux-en-Y gastric bypass

There were significantly more complications in Group I (11.4%) compared to Group II (6.6%) (*p* = 0.022; Table 4). There was one (0.7%) mortality in Group I and eight (0.1%) in Group II (*p* = 0.17 on Fisher’s exact test). Additionally, 38 (0.5%) patients in Group II had symptomatic COVID-19 infection within 30 days of the surgical operation compared to none in Group I (*p* = 1.000).

**Table 4:** Comparison between the two age groups according to the outcome parameters

	Group I (≥ 65) (*n* =149)	Group II (˂ 65) (*n* =6923)	*χ*^2^	*p*
Complications				
No	132 (88.6%)	6463 (93.4%)	5.265^*^	0.022^*^
Yes	17 (11.4%)	460 (6.6%)		
Clavien-Dindo (CD) Score				
0	132 (88.6%)	6463 (93.4%)		
1	3 (2%)	162 (2.3%)		
2	4 (2.7%)	132 (1.9%)		
3A	4 (2.7%)	29 (0.4%)		
3B	4 (2.7%)	91 (1.3%)		
4A	1 (0.7%)	31 (0.4%)		
4B	0 (0%)	7 (0.1%)		
5 (Mortality)	1 (0.7%)	8 (0.1%)		
COVID within 30 days				
No	149 (100%)	6885 (99.5%)	0.822	^FE^p= 1.000
Yes	0 (0%)	38 (0.5%)		

χ^2^: Chi-square test; FE: Fisher Exact; p: *p*-value for comparing the two studied groups; *Statistically significant at *p* ≤ 0.05

*CD*, Clavein-Dindo Score; *COVID*, Novel Coronavirus 2019

Table 5 presents 30-day morbidity and mortality analysed by procedure type in both groups. Differences in morbidity and mortality were only significant for LSG.

**Table 5:** Morbidity and Mortality rates in each group sub-divided by procedure type

		Overall	Group I	Group II	*p*-value
Total patients		(*n* = 7072)	(*n* = 149)	(*n* = 6923)	CD grade as a binary variable
LSG	30-day Morbidity	233/3983 (5.8%)	12/76 (15.8%)	221/3907 (5.7%)	< 0.001
	30-day mortality	4/3983 (0.10%)	1/76 (1.32%)	3/3907 (0.08%)	
RYGB	30-day Morbidity	166/2087 (8.0%)	4/49 (8.2%)	162/2038 (7.9%)	0.956
	30-day mortality	0	0	0	
OAGB	30-day Morbidity	53/702 (7.5%)	0/13	53/689 (7.7%)	0.298
	30-day mortality	3/702 (0.43%)	0	3/689 (0.44%)	
Other	30-day Morbidity	25/300 8.3%	1/11 (9.1%)	24/289 (8.3%)	0.926
	30-day mortality	2/300 (0.67%)	0	2/289 (0.69%)	

*LSG*, laparoscopic sleeve gastrectomy; *OAGB*, one anastomosis gastric bypass; *RYGB*, Roux-en-Y gastric bypass

Chi-square test performed (age more than 65 compared against presence/ absence of morbidity/ mortality)

## Discussion

This study has demonstrated that 30-day morbidity was significantly higher for patients ≥ 65 years of age receiving bariatric surgery compared to those < 65 years of age during the COVID-19 pandemic. However, there was no significant difference in 30-day mortality or 30-day symptomatic postoperative COVID-19 infection rates between the two groups.

The finding of increased 30-day morbidity in patients ≥ 65 years old maybe because 94.6% of patients in Group I had at least one co-morbidity compared to 68.3% in Group II. This is similar to the findings by Susmallian et al. who identified that 77% of patients ≥ 65 years of age had at least one comorbidity [7]. Similarly, Bhandari et al. demonstrated that 47.3%, 84.2%, and 17.9% of patients ≥ 65 years old suffered from diabetes, hypertension, and coronary heart disease compared to 20.1%, 23.4%, and 3.8%, respectively, in the younger age group [19].

The 30-day morbidity in our series was significantly higher in the older patients at 11.4% compared to 6.6% in the younger age group. A previous analysis of the National Surgical Quality Improvement Program (NSQIP) database demonstrated similar findings with increased rates of serious morbidity in the older age group compared to younger patients [20]. Another study reported a higher overall complication rate of 8.42% in older patients compared to 5.59% in the younger group, with significant differences in CD grades 3B and 4A [7].

In the current study, there was one mortality in the older age group (*n* = 149) and eight in the younger group (*n* = 6923), representing 0.7% and 0.1%, respectively. Though the difference did not reach statistical significance, this may be due to the small sample size. Bariatric teams should, therefore, be careful in offering bariatric surgery to patients in this age group. In contrast, a recent meta-analysis found that the mortality rate after LSG was similar at 0.2% in patients > 60 years and those ≤ 60 years of age whereas the mortality after RYGB was 2.2% and 0% (0/182), respectively [21]. However, those authors used 60 years as the cutoff which is no longer used to define older patients by WHO and other such bodies.

LSG was unsurprisingly the most commonly performed operation type in both groups (Group 1: 51.0%; Group 2: 56.4%) (Table 3). Importantly, there were no significant differences in procedure choice between the two groups. Although the 30-day morbidity was significantly higher for LSG with Group I (15.8% vs 5.7%), it is difficult to draw firm conclusions from this due to the relatively low patient numbers (*n* = 76). All other procedures had comparable morbidity rates in both groups but once again there is potential for type II error due to small numbers. We cannot make any justifiable conclusions regarding morbidity and mortality of different procedures in two groups based on the data in our study. Authors would however suggest that procedure selection is made in the usual manner on an individualised basis for each patient taking into account their wishes and specific characteristics.

### Strengths and Weaknesses

This study has several weaknesses. Firstly, it only included data from participating centres and might therefore not represent the complete global picture. Additionally, we cannot guarantee that all contributors submitted all their consecutive patients during the study period, though collaborators were repeatedly reminded to do so. It is also possible that all adverse outcomes were not reported, but it is hoped that anonymous data collection and reporting would have discouraged any underreporting. Lastly, there were only 149 patients in the older age group, meaning that there is a potential for Type II error concerning the difference in mortality which indeed appears to be higher in the older population (0.7% vs 0.1%).

At the same time, this is the first international study examining the safety of bariatric surgery in those ≥ 65 years of age during the COVID-19 pandemic, which is known to have affected older people disproportionately. Moreover, this study included a broad range of patients representing a wide range of demographics, geographical distribution, stage of COVID-19 pandemic severity in the host population, and surgeon and centre experiences. Other strengths of this study include the large sample size, the global reach of the study, high data completion rate, and nearly complete follow-up.

## Conclusion

Bariatric surgery during the COVID-19 pandemic was associated with higher 30-day morbidity in older patients (≥ 65 years old) compared to younger patients. The mortality and postoperative COVID-19 infection rates were comparable to the younger age group.

### Author Contribution

RS: conceptualization, methodology, investigation, formal analysis. IO: formal analysis, writing — original draft preparation, discussion of the results, writing — review & editing. BM, YR, YG, AAT, CL, and TW: investigation, data curation. KM: conceptualization, methodology, writing — review & editing, supervision. All authors have seen the final manuscript and approved it. The study was designed and conducted by the study group and the authors on behalf of GENEVA collaborators.

## Data Availability

The data used to support the findings of this study can be released upon request.
