# Correction to: Safety of Bariatric Surgery in ≥ 65-Year-Old Patients during the COVID-19 Pandemic

**DOI:** 10.1007/s11695-026-08493-9

**Published:** 2026-01-19

**Authors:** Rishi Singhal, Islam Omar, Brijesh Madhok, Yashasvi Rajeev, Yitka Graham, Abd A. Tahrani, Christian Ludwig, Tom Wiggins, Kamal Mahawar

**Affiliations:** 1 Upper GI unit, Birmingham Heartlands Hospital, University Hospital Birmingham NHS Foundation Trust, Bordesley Green East, Birmingham, West Midlands B9 5SS UK; 2 Healthier Weight, Birmingham, UK; 3 General Surgery Department, Wirral University Teaching Hospital NHS Foundation Trust, North West, Wirral, UK; 4 Upper GI unit, University Hospital of Derby and Burton NHS Foundation Trust, East Midlands, Derby, UK; 5 Pediatric Accidents and Emergencies Department, London Northwest University Healthcare NHS Trust, London, UK; 6 Faculty of Health Sciences and Wellbeing, University of Sunderland, North East, Sunderland, UK; 7 Faculdad de Pyscologia, Universidad Anahuac, Anahuac, Naucalpan de Juárez, Mexico; 8 Institute of Metabolism and Systems Research, College of Medical and Dental Sciences, University of Birmingham, Birmingham, UK; 9 Centre for Endocrinology, Diabetes, and Metabolism, Birmingham Health Partners, Birmingham, UK; 10 Department of Diabetes and Endocrinology, University Hospitals Birmingham NHS Foundation Trust, Birmingham, West Midlands UK; 11 Institute of Metabolism and Systems Research, College of Medical and Dental Sciences, University of Birmingham, Birmingham, West Midlands UK; 12 Bariatric Unit, South Tyneside and Sunderland NHS Foundation Trust, North East, Sunderland, UK

**Correction to: Obesity Surgery (2022) 32:1-13**

10.1007/s11695-022-06067-z

The name of Geneva Collaborator 141 is incorrect. The correct name is Ahmet Bozdag.

